# Glucosylsphingosine (Lyso-Gb1) Dynamics in Untreated States in Gaucher Disease

**DOI:** 10.3390/ijms27093726

**Published:** 2026-04-22

**Authors:** Tama Dinur, Peter Bauer, Sabine Schroeder, Guido Kramp, Christian Beetz, Michal Becker-Cohen, Majdolen Istaiti, Dafna Frydman, Elena Shulman, Ari Zimran, Shoshana Revel-Vilk

**Affiliations:** 1 Gaucher Unit, The Eisenberg R&D Authority, Shaare Zedek Medical Center, Jerusalem 9103102, Israel; dinurtama@gmail.com (T.D.); michalbc@szmc.org.il (M.B.-C.); majdolenist@gmail.com (M.I.); dafnaf@szmc.org.il (D.F.); elename@szmc.org.il (E.S.);; 2 Centogene GmbH, 18055 Rostock, Germany; peter.bauer@centogene.com (P.B.); sabine.schroeder@centogene.com (S.S.); guido.kramp@centogene.com (G.K.); christian.beetz@centogene.com (C.B.); 3 Agyany Pharma Ltd., Jerusalem 9695614, Israel; 4 Faculty of Medicine, Hebrew University of Jerusalem, Jerusalem 9112002, Israel

**Keywords:** glucosylsphingosine, lyso-Gb1, Gaucher disease, enzyme replacement therapy (ERT), substrate reduction therapy (SRT)

## Abstract

Glucosylsphingosine (lyso-Gb1) serves as a biomarker for evaluating disease activity in Gaucher disease (GD). While treatment-related changes are documented, the dynamics of lyso-Gb1 during untreated states remain poorly understood. This retrospective, longitudinal cohort study utilized a large GD database comprising 701 patients and over 6200 visits with lyso-Gb1 measurements. Patients with at least two untreated visits were included in the analysis (n = 272). A significant change was defined as ≥50 ng/mL for lyso-Gb1, ≥1 g/dL for hemoglobin, and ≥50 × 10^9^/L for platelet count. Multivariable logistic regression analyses identified clinical factors associated with lyso-Gb1 decline or an increase. During untreated states, 35 patients (12.9%; 95% CI 9.4–17.5%) exhibited a decline in lyso-Gb1, with a median decrease of 96.3 ng/mL. This decline was more common in females (OR 3.50, *p* = 0.032) and associated with higher initial lyso-Gb1 levels (*p* < 0.001) and baseline hemoglobin (*p* = 0.032). An increase in lyso-Gb1 was observed in 98 patients (36.0%; 95% CI 30.5–41.9%), with a median rise of 135.1 ng/mL. This increase correlated with lower baseline platelet counts (*p* = 0.003), lower baseline hemoglobin (*p* = 0.002), and longer follow-up duration (*p* = 0.001). In many cases, lyso-Gb1 increases were observed without a preceding change in hemoglobin or platelet count. In summary, declines in lyso-Gb1 in untreated states are rare but possible. The association with female sex may reflect inflammatory effects. Although increases in lyso-Gb1 were expected without treatment, they occurred mainly in patients with higher disease severity markers. Nevertheless, most patients in the untreated states remained stable within ±50 ng/mL. These findings demonstrate a heterogeneous trajectory of lyso-Gb1 across untreated states and highlight the importance of interpreting lyso-Gb1 changes within the clinical context when making treatment decisions.

## 1. Introduction

Gaucher disease (GD) is the most common lysosomal storage disorder and results from biallelic pathogenic variants in the *GBA*1 gene, leading to the deficient activity of the lysosomal enzyme β-glucocerebrosidase [1]. The enzymatic defect causes a progressive accumulation of glucosylceramide and related sphingolipids within macrophages, leading to the characteristic “Gaucher cells” that infiltrate the spleen, liver, bone marrow, and other tissues. Although enzyme replacement and substrate reduction therapies have substantially altered disease outcomes, some patients with GD remain untreated [2,3,4].

Glucosylsphingosine (lyso-Gb1), the deacylated derivative of glucosylceramide, has emerged as a highly sensitive and specific biomarker for GD [5,6,7,8], reflecting the underlying accumulation of glucosylceramide and the metabolic activity of Gaucher macrophages. Plasma lyso-Gb1 concentrations are markedly elevated in untreated patients and typically decline following therapy initiation, correlating with disease burden and treatment response [9,10,11,12,13]. Compared with traditional biomarkers such as chitotriosidase, lyso-Gb1 more directly reflects the underlying metabolic defect and has increasingly become central to diagnostic and monitoring strategies.

The clinical course of untreated GD is characterized by substantial inter-individual variability, ranging from stable mild phenotypes to progressive hematologic and skeletal complications. In clinical practice, decisions regarding treatment initiation frequently incorporate longitudinal trends in biomarkers, including lyso-Gb1 [14,15]. However, the interpretation of lyso-Gb1 changes in untreated patients remains challenging, particularly in individuals identified through screening or with mild disease, for whom treatment decisions are less straightforward.

Most existing data describing lyso-Gb1 dynamics derive from treated cohorts or short-term observations, and the natural behavior during untreated follow-up remains incompletely defined. Specifically, the frequency and magnitude of spontaneous fluctuations, as well as the clinical factors associated with these changes, are not well established. Therefore, the aim of this retrospective, longitudinal study was to characterize lyso-Gb1 trajectories during untreated states and to identify clinical factors associated with lyso-Gb1 increases or decreases. Focusing on untreated states, rather than never-treated status, allowed us to assess lyso-Gb1 behavior during periods without therapy across real-world longitudinal follow-ups.

## 2. Results

### 2.1. Study Cohort

Among 701 patients with longitudinal data, 272 met the inclusion criteria for analysis in untreated states (Table 1). Compared with treated individuals, patients with untreated states were younger at study entry and at the last visit, were more frequently classified as having a mild genotype, and had a slightly shorter follow-up time. Hematologic parameters were broadly similar between groups. Lyso-Gb1 levels were similar at study entry but significantly lower in the treated cohort at the last visit.

### 2.2. Lyso-Gb1 Trends During Untreated States

**Figure 1:**
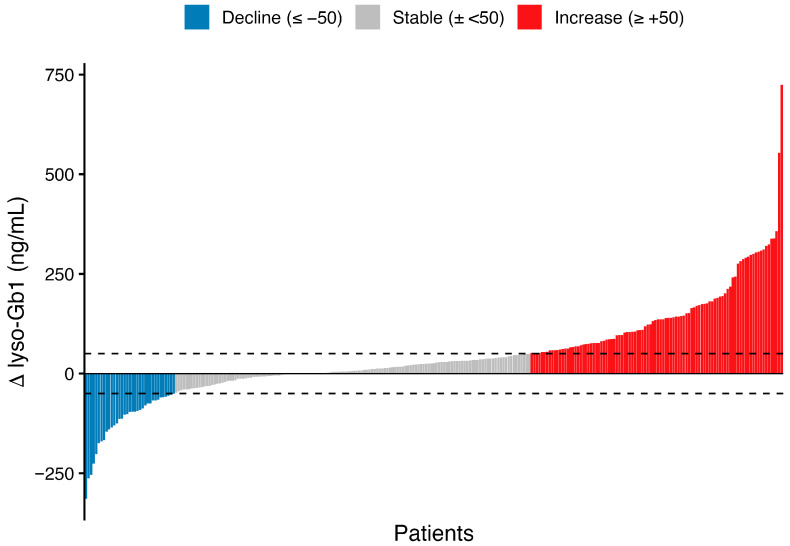
The individual change in lyso-Gb1 during untreated states. Each bar represents one patient and shows the change in plasma lyso-Gb1 between the first and last untreated measurements (Δ lyso-Gb1 = last−first), with patients ordered from the largest decrease to the largest increase. Dashed horizontal lines indicate the predefined thresholds of −50 ng/mL and +50 ng/mL. Bars are colored by category: decline (≤−50 ng/mL), stable (within ±50 ng/mL), and increase (≥+50 ng/mL).

During untreated states, 35 patients (12.9%; 95% CI, 9.4–17.5%) experienced a decline in lyso-Gb1 concentrations (≥50 ng/mL), with a median decrease of 96.3 ng/mL; 17 had declines exceeding 100 ng/mL. Increases in lyso-Gb1 were more common, occurring in 98 patients (36.0%; 95% CI 30.5–41.9%), with a median rise of 135.1 ng/mL; 62 demonstrated an increase greater than 100 ng/mL. Overall, approximately half of the patients in the untreated states remained within the predefined ±50 ng/mL stability threshold over time. The distribution of longitudinal changes is shown in Figure 1. Representative longitudinal trajectories for selected patients in the decline, stable, and increase groups are shown in Supplementary Figure S1A–C.

Monitoring intensity during the untreated states was comparable across the decline, increase, and stable groups, with no significant differences in the number of measurements per patient (Table 2). Follow-up intervals differed modestly across groups, with a shorter interval observed in the decline group compared with the increase group (*p* = 0.036), whereas other pairwise comparisons were not significant. Among patients with exactly two measurements, neither the proportion of such patients nor the interval between measurements differed between groups.

### 2.3. Factors Associated with Lyso-Gb1 Decline

**Figure 2:**
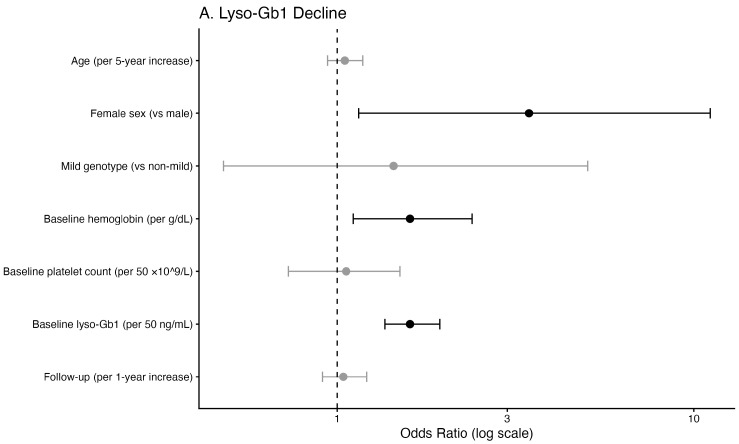

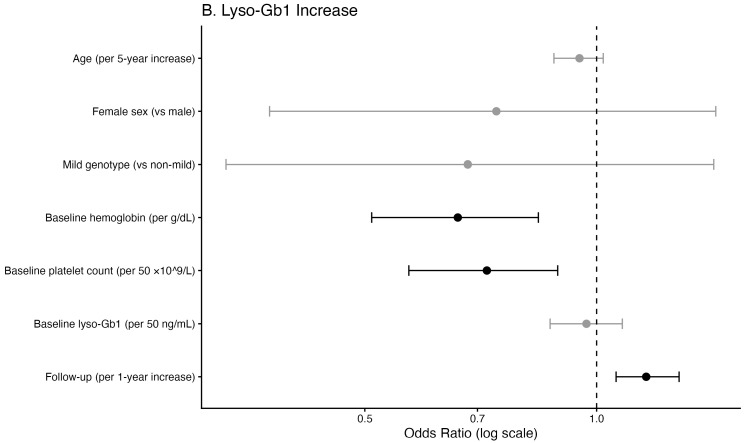
Multivariable logistic regression models for significant lyso-Gb1 decline (**A**) and increase (**B**) during untreated states. Points represent odds ratios, and horizontal lines represent 95% confidence intervals. The dashed vertical line indicates OR = 1.0. Black markers denote statistically significant associations (*p*-value < 0.05).

Multivariable logistic regression analysis identified female sex as independently associated with increased odds of significant lyso-Gb1 decline during untreated follow-up (Table 3; Figure 2A). Higher baseline hemoglobin and higher baseline lyso-Gb1 levels were also independently associated with decline. Age, genotype severity, baseline platelet count, and duration of untreated follow-up were not significantly associated with decreasing lyso-Gb1 levels.

### 2.4. Factors Associated with Lyso-Gb1 Increase

Significant increases in lyso-Gb1 were primarily associated with lower baseline platelet count, lower baseline hemoglobin levels, and a longer duration of untreated follow-up (Table 3; Figure 2B). No significant association was observed between lyso-Gb1 trends and concurrent changes in hemoglobin or platelet count.

Among patients with increasing lyso-Gb1, hemoglobin decline was documented before lyso-Gb1 increase in only eight cases (8%), while platelet decline was documented before lyso-Gb1 increase in four cases (4%). In most patients, hematologic deterioration occurred after the lyso-Gb1 rise or at the same visit or was not observed during follow-up.

### 2.5. Analysis of Never-Treated Patients

We performed an additional analysis restricted to 543 patients who were never treated and compared them with those who were always treated throughout follow-up (Table 4). In this stricter comparison, never-treated patients were younger, had a substantially higher proportion of a mild genotype, had shorter follow-up, and had higher last lyso-Gb1 values than always-treated patients.

Among patients who were never treated, 20 (8.1%; 95% CI 5.3–12.2%) experienced a decline in lyso-Gb1 concentrations (≥50 ng/mL), with a median decrease of 96.2 ng/mL; 9 had declines exceeding 100 ng/mL. Increases in lyso-Gb1 were more frequent, occurring in 63 patients (25.6%; 95% CI 20.6–31.4%), with a median rise of 132.0 ng/mL; 39 showed an increase greater than 100 ng/mL. Overall, most patients who were never treated (66.3%) remained within the predefined ±50 ng/mL stability threshold over time. The distribution of longitudinal changes in patients who were never treated is shown in Supplementary Figure S2.

In the multivariable logistic regression analysis restricted to patients who were never treated, older age, higher baseline lyso-Gb1 levels, and longer follow-up duration were independently associated with increased odds of a significant lyso-Gb1 decline, whereas significant increases in lyso-Gb1 were independently associated with younger age, lower baseline platelet count, and longer follow-up duration (Table 5).

## 3. Discussion

This large, retrospective, longitudinal study demonstrates that lyso-Gb1 trajectories during untreated states in GD are heterogeneous rather than uniformly progressive. Although increases in lyso-Gb1 were observed in approximately one-third of patients, nearly half of the cohort remained stable within a predefined range during prolonged untreated follow-up, and a minority of patients exhibited spontaneous declines. These findings highlight that biochemical variability can occur even in the absence of treatment and emphasize that changes in lyso-Gb1 should be interpreted cautiously within the broader clinical context; the reliable prediction of increases or declines at the individual patient level is not always possible.

Our findings extend observations from natural history studies demonstrating that untreated GD can follow a heterogeneous clinical course, with some patients remaining stable for extended periods, while others experience progressive disease [4,16,17]. In this context, the stability observed in most patients who were never treated suggests that biochemical progression is not inevitable during untreated follow-up, particularly in individuals with milder disease phenotypes.

In contrast, 36% of patients showed an increase in lyso-Gb1 levels during the untreated states. These increases were independently linked to lower baseline platelet counts and hemoglobin levels, both indicators of more severe disease. This finding aligns with previous studies showing correlations between lyso-Gb1 levels and clinical disease burden [18,19]. A longer follow-up duration was also associated with a higher chance of lyso-Gb1 increase, supporting the concept that GD may progress over time and that routine follow-up visits are warranted [20,21,22]. In many patients, elevations in lyso-Gb1 were observed without a preceding measurable decline in hematologic parameters, suggesting that lyso-Gb1 may reflect early metabolic changes before overt hematologic progression becomes apparent [23]. However, increases in lyso-Gb1 do not necessarily indicate immediate clinical worsening. In practice, rising lyso-Gb1 levels should lead to closer monitoring and re-evaluation, often including repeat testing at an earlier time, rather than immediate treatment unless other clinical signs are present [17]. In stable patients not receiving GD-specific therapy, lyso-Gb1 may be assessed as part of routine follow-up, often every 6–12 months. When a considerable rise is observed or when there is concern for clinical instability, reassessment may be considered even within 3 months, depending on the overall clinical context.

A notable finding of this study is that a minority of patients experienced substantial declines in lyso-Gb1 during follow-up without treatment. Although uncommon, these reductions were sometimes large, exceeding 100 ng/mL in several patients. In the broader analysis across untreated states, female sex was independently associated with a higher likelihood of decline in lyso-Gb1. However, this observation should be interpreted cautiously, as it was based on a relatively small number of events and was not observed in the restricted never-treated analysis. While sex-related differences in immune responses and inflammatory signaling have been described in other lysosomal and metabolic disorders [24,25,26], this mechanism was not directly tested in the present study. Accordingly, this finding should be regarded as hypothesis-generating and in need of further evaluation. Higher baseline lyso-Gb1 levels were also associated with subsequent decreases, which may partly reflect regression toward the mean, whereby individuals with particularly elevated baseline values demonstrate reductions on repeat testing [27]. Because lyso-Gb1 reflects macrophage activation and sphingolipid turnover, transient metabolic or inflammatory fluctuations may also contribute to temporary elevations followed by spontaneous declines. Although baseline hemoglobin was associated with lyso-Gb1 decline in multivariable analysis, descriptive comparisons showed largely overlapping hemoglobin distributions between groups, suggesting that the association reflects a modest continuous effect captured by the regression model rather than a clinically meaningful difference in baseline hemoglobin levels.

Interestingly, in the restricted analysis of patients who were never treated, older age was associated with lyso-Gb1 decline, whereas younger age was associated with lyso-Gb1 increase. One possible explanation is that patients who were never treated at an older age represent a selected group with long-term stable or milder disease, in whom downward fluctuations or gradual reductions in lyso-Gb1 may be more likely [3]. In contrast, younger patients who were never treated may still be earlier in their disease course and therefore more likely to show an increase in lyso-Gb1 over time [16].

Importantly, the threshold used to define a change in lyso-Gb1 in this study was intentionally conservative. We predefined a change as an absolute increase or decrease of at least ±50 ng/mL. This threshold was selected to account for biological variability in lyso-Gb1 measurements while capturing changes that would realistically influence clinical management. Analytical variability in the DBS-based lyso-Gb1 assay is low, with reported intra-assay coefficients of variation below 6% across clinically relevant concentrations, indicating high measurement precision [23]. Nevertheless, smaller fluctuations may occur due to physiological variation, intercurrent illness, or other transient factors and may not necessarily reflect true changes in disease activity. Even when lyso-Gb1 changes exceed this threshold, lyso-Gb1 trends should be interpreted in conjunction with clinical findings, including hematologic parameters, organ involvement, skeletal disease, and overall disease trajectory, rather than being used in isolation for treatment decisions [17].

Several limitations should be considered when interpreting these findings. First, the retrospective design limits the ability to control for potential confounders such as intercurrent infections, inflammatory conditions, hormonal changes, or other metabolic factors that may influence sphingolipid metabolism and lyso-Gb1 levels. Second, patients in untreated states in this cohort were more likely to have milder genotypes and disease manifestations, which may limit the generalizability of these findings to patients with more severe disease. Third, visit intervals were not standardized and varied among patients, which may influence the magnitude of observed lyso-Gb1 changes. Some patients had only two measurements, which may increase the risk of misclassification of longitudinal trends and introduce potential bias. However, the interval between these two measurements did not differ between groups, suggesting that any potential misclassification is likely non-differential and unlikely to substantially bias the observed associations. Finally, although hematologic parameters were evaluated, other clinically important manifestations of GD, including skeletal disease and organ volumes, were not incorporated into the longitudinal models.

## 4. Materials and Methods

### 4.1. Study Design and Data Source

This retrospective, longitudinal cohort study used data from the Gaucher Unit database at Shaare Zedek Medical Center, Jerusalem, Israel. The institutional database integrates demographic, clinical, treatment, laboratory, and lyso-Gb1 data from patients with GD. Lyso-Gb1 quantification was performed from dried blood spot (DBS) samples at Centogene GmbH (Rostock, Germany), as previously described [7]. Demographic information was merged with longitudinal treatment records, plasma lyso-Gb1 measurements, and hematologic laboratory values. Laboratory values were matched to lyso-Gb1 measurements within a ±30-day window, and when duplicate laboratory entries were present, standardized values were prioritized. The definitions of mild versus severe genotypes were determined for type 1 GD by N370S (c.1226A > G) homozygous, and N370S/R496H (c.1604G) compound heterozygous genotypes were categorized as “mild”, whereas all other genotypes were categorized as “severe”.

### 4.2. Definition of Untreated States and Longitudinal Assessment

The study population comprised patients with untreated states, including individuals with a mild phenotype not requiring GD-specific therapy, those who were eventually treated but contributed data prior to treatment initiation, and those with at least 365 days of follow-up after treatment discontinuation without the reinitiation of therapy. Patients were eligible for inclusion in the untreated longitudinal analysis if they had at least two separate visits during untreated states with available lyso-Gb1 measurements. For each patient, baseline was defined as the first untreated lyso-Gb1 measurement, and longitudinal change was assessed between the first and last untreated visits. Given the retrospective nature of this study, the number of measurements and the intervals between visits were not standardized across patients.

The longitudinal change in lyso-Gb1 was calculated as the difference between the last and first untreated measurements. An absolute increase or decrease of at least 50 ng/mL was predefined as the threshold for classifying directional change. Patients were categorized as having a significant decrease, a significant increase, or stable levels within ±50 ng/mL. Changes in hemoglobin were considered clinically meaningful if ≥1 g/dL and changes in platelet count were if ≥50 × 10^9^/L.

### 4.3. Statistical Analysis

Continuous variables are presented as medians with interquartile ranges (Q1–Q3) and categorical variables as counts and percentages. Comparisons between treated and untreated groups were performed using the Wilcoxon rank-sum test for continuous variables and Pearson’s chi-squared test for categorical variables. The proportions of patients demonstrating significant decreases, increases, or stability in lyso-Gb1 during untreated follow-up were estimated, along with corresponding 95% confidence intervals. Supplementary spaghetti plots were generated for 20 patients in each group. Patients in the stable group were randomly selected, whereas patients in the decline and increase groups were selected from those with absolute net first-to-last lyso-Gb1 changes greater than 100 ng/mL.

For analyses of monitoring intensity during untreated follow-up, differences in the number of lyso-Gb1 measurements per patient and in the interval between the first and last measurements across the decline, stable, and increase groups were assessed using Kruskal–Wallis tests. Pairwise group comparisons for the follow-up interval were performed using Wilcoxon rank-sum tests with Holm adjustment for multiple testing. The same analyses were repeated in the subset of patients with two measurements.

To identify clinical factors associated with longitudinal lyso-Gb1 dynamics, two separate multivariable logistic regression models were constructed: one to model a significant decrease and the other to model a significant increase. Covariates included age (per 5-year increase), sex, genotype severity (mild vs. non-mild), baseline hemoglobin (per g/dL), baseline platelet count (per 50 × 10^9^/L), baseline lyso-Gb1 (per 50 ng/mL), and duration of untreated follow-up (per 1-year increase). Adjusted odds ratios with 95% confidence intervals were reported. All tests were two-sided, and a *p*-value < 0.05 was considered statistically significant. Statistical analyses were performed using R software (version 4.5.2; R Foundation for Statistical Computing, Vienna, Austria).

## 5. Conclusions

In this large longitudinal cohort of patients with GD during untreated states, lyso-Gb1 trajectories were heterogeneous rather than consistently progressive. Although increases in lyso-Gb1 were common and linked with markers of higher disease burden, such as lower platelet and hemoglobin levels, in nearly half of the patients, lyso-Gb1 remained stable in the untreated state. A smaller group of patients showed spontaneous declines in lyso-Gb1, indicating that fluctuations can happen even without therapy. Importantly, increases in lyso-Gb1 were often observed without a preceding measurable hematologic decline, supporting its value as an early biomarker of disease activity. These results support using lyso-Gb1 as a sensitive biomarker for monitoring untreated states but highlight that isolated changes should not automatically prompt treatment. Instead, lyso-Gb1 trends should be evaluated alongside clinical assessments, hematologic parameters, and other disease signs. Overall, this study offers real-world insights into lyso-Gb1 variation during untreated states in GD and highlights the importance of longitudinal assessment and individualized clinical interpretation when using this biomarker to inform management decisions.

## Figures and Tables

**Table 1:** Characteristics of patients with Gaucher disease during treated and untreated states.

	Treated States	Untreated States	*p*-Value
*n*	429	272	
Female, *n* (%)	227, 54%	152, 56%	0.5
Mild genotype, *n* (%)	235, 55%	214, 79%	<0.001
Follow-up, years: median (Q1–Q3)	7.7 (2.6–10.1)	6.4 (3.5–8.7)	0.02
Age, years: median (Q1–Q3)			
First	38.8 (21.5–52.8)	29.9 (13.1–47.5)	<0.001
Last	45.8 (27.4–60.0)	34.1 (19.3–55.0)	<0.001
Hemoglobin, g/dL: median (Q1–Q3)			
First	13.2 (12.0–14.4)	12.9 (12.0–14.0)	0.069
Last	13.4 (12.2–14.6)	13.3 (12.3–14.3)	0.6
Platelet count, ×10^9^/L: median (Q1–Q3)			
First	146 (109–199)	149 (105–209)	0.7
Last	172 (129–224)	166 (123–212)	0.076
Lyso-Gb1, ng/mL: median (Q1–Q3)			
First	105 (44–253)	119 (52–243)	0.3
Last	86 (35–188)	124 (50–238)	<0.001

Wilcoxon rank-sum test for continuous variables; Pearson’s chi-squared test for categorical variables. The definition of mild vs. severe *GBA1* genotypes is detailed in Section 4.

**Table 2:** Lyso-Gb1 testing across trend groups.

Group	Total Tests	Test Per Patient	Interval, Years *	Patients with Two Tests	Interval, Year *
Decline	146	3.0 (2–20)	2.34 (0.84–6.82)	14	0.73 (0.08–3.23)
Increase	531	4.0 (2–16)	5.2 (2.61–8.11)	25	1.08 (0.56–3.5)
Stable	618	3.5 (2–18)	4.47 (1.32–7.52)	47	1.06 (0.49–2.39)

* median (IQR).

**Table 3:** Multivariable logistic regression analysis of factors associated with lyso-Gb1 decline or increase during untreated states.

Variable	Decline OR (95% CI)	*p*-Value	Increase OR (95% CI)	*p*-Value
Age (per 5-year increase)	1.05 (0.94–1.18)	0.368	0.95 (0.88–1.02)	0.184
Female (vs. male)	3.50 (1.17–11.79)	0.032	0.73 (0.37–1.43)	0.364
Mild genotype (vs. non-mild)	1.41 (0.47–4.97)	0.562	0.66 (0.32–1.38)	0.269
Baseline hemoglobin (per g/dL)	1.52 (1.05–2.26)	0.032	0.67 (0.52–0.85)	0.002
Baseline platelet count (per 50 × 10^9^/L)	1.11 (0.76–1.56)	0.581	0.72 (0.57–0.89)	0.003
Baseline lyso-Gb1 (per 50 ng/mL)	1.60 (1.36–1.94)	<0.001	0.98 (0.88–1.08)	0.671
Follow-up (per 1-year increase)	1.06 (0.92–1.23)	0.445	1.17 (1.07–1.29)	0.001

**Table 4:** Characteristics of patients who were always and never treated.

	Always Treated	Never Treated	*p*-Value
*n*	297	246	
Female, *n* (%)	161 (54%)	123 (51%)	0.4
Mild genotype, *n* (%)	143 (48%)	206 (84%)	<0.001
Follow-up, years: median (Q1–Q3)	9.1 (6.7–10.3)	4.2 (0.0–8.0)	<0.001
Age, years: median (Q1–Q3)			
First	40.2 (26.9–54.7)	29.8 (13.8–51.0)	<0.001
Last	48.6 (34.1–63.1)	32.8 (18.8–55.3)	<0.001
Hemoglobin, g/dL: median (Q1–Q3)			
First	13.4 (12.3–14.5)	13.2 (12.2–14.2)	0.094
Last	13.6 (12.4–14.7)	13.3 (12.4–14.4)	0.2
Platelet count, ×10^9^/L: median (Q1–Q3)			
First	154 (121–208)	161 (119–220)	0.4
Last	182 (142–227)	164 (119–212)	0.006
Lyso-Gb1, ng/mL: median (Q1–Q3)			
First	81 (40–175)	107 (41–225)	0.084
Last	69 (34–157)	131 (52–267)	<0.001

Wilcoxon rank-sum test for continuous variables; Pearson’s chi-squared test for categorical variables. The definition of mild vs. severe *GBA1* genotypes is detailed in Section 4.

**Table 5:** Multivariable logistic regression analysis of factors associated with lyso-Gb1 decline or increase in lyso-Gb1 in never-treated individuals.

Variable	Decline OR (95% CI)	*p*-Value	Increase OR (95% CI)	*p*-Value
Age (per 5-year increase)	1.11 (1.03–1.19)	0.006	0.86 (0.80–0.91)	<0.001
Female (vs. male)	1.07 (0.57–2.01)	0.841	1.01 (0.61–1.67)	0.962
Mild genotype (vs. non-mild)	1.25 (0.69–2.32)	0.470	1.18 (0.74–1.91)	0.483
Baseline hemoglobin (per g/dL)	1.02 (0.84–1.22)	0.873	0.90 (0.76–1.04)	0.163
Baseline platelet count (per 50 × 10^9^/L)	1.00 (0.83–1.20)	0.976	0.77 (0.65–0.90)	0.002
Baseline lyso-Gb1 (per 50 ng/mL)	1.66 (1.49–1.87)	<0.001	1.07 (0.98–1.16)	0.107
Follow-up (per 1-year increase)	1.24 (1.14–1.37)	<0.001	1.13 (1.06–1.22)	<0.001

## Data Availability

Data cannot be shared due to ethical and privacy issues. Access to deidentified data may be considered upon reasonable request, subject to institutional approval and in compliance with ethical and privacy regulations.

## Supplementary Materials

The following supporting information can be downloaded at https://www.mdpi.com/article/10.3390/ijms27093726/s1.
