# Supplementary material for: Glucosylsphingosine (Lyso-Gb1) Dynamics in Untreated States in Gaucher Disease
Source: Int J Mol Sci. 2026 Apr 22;27(9):3726. doi: 10.3390/ijms27093726 (PMC13163878; doi:10.3390/ijms27093726)
Supplement: Supplementary file 1 [file ijms-27-03726-s001.zip › Supplementary Figure legends.pdf]

Supplementary Figure S1. Representative longitudinal lyso-Gb1 trajectories during untreated states. Supplementary Figure 1. Representative longitudinal lyso-Gb1 trajectories during untreated follow-up. (A) Decline group, (B) increase group, and (C) stable group. Each line represents one patient and includes all available untreated lyso-Gb1 measurements within the same untreated interval used for classification in the main analysis. Twenty patients were included in each panel. Patients in the stable group were randomly selected, whereas patients in the decline and increase groups were selected from those with net first-to-last lyso-Gb1 changes greater than 100 ng/mL in absolute value to better illustrate representative trajectories.

Supplementary Figure S2. Individual change in lyso-Gb1 for patients who were never treated. Each bar represents one patient and shows the change in lyso-Gb1 between the first and last untreated measurements (Δ lyso-Gb1 = last − first), with patients ordered from the largest decrease to the largest increase. Dashed horizontal lines indicate the predefined thresholds of −50 ng/mL and +50 ng/mL. Bars are colored by category: decline (≤ −50 ng/mL), stable (within ±50 ng/mL), and increase (≥ +50 ng/mL).
