# Supplementary figures and images for: Glucosylsphingosine (Lyso-Gb1) Dynamics in Untreated States in Gaucher Disease
Source: Int J Mol Sci. 2026 Apr 22;27(9):3726. doi: 10.3390/ijms27093726 (PMC13163878; doi:10.3390/ijms27093726)

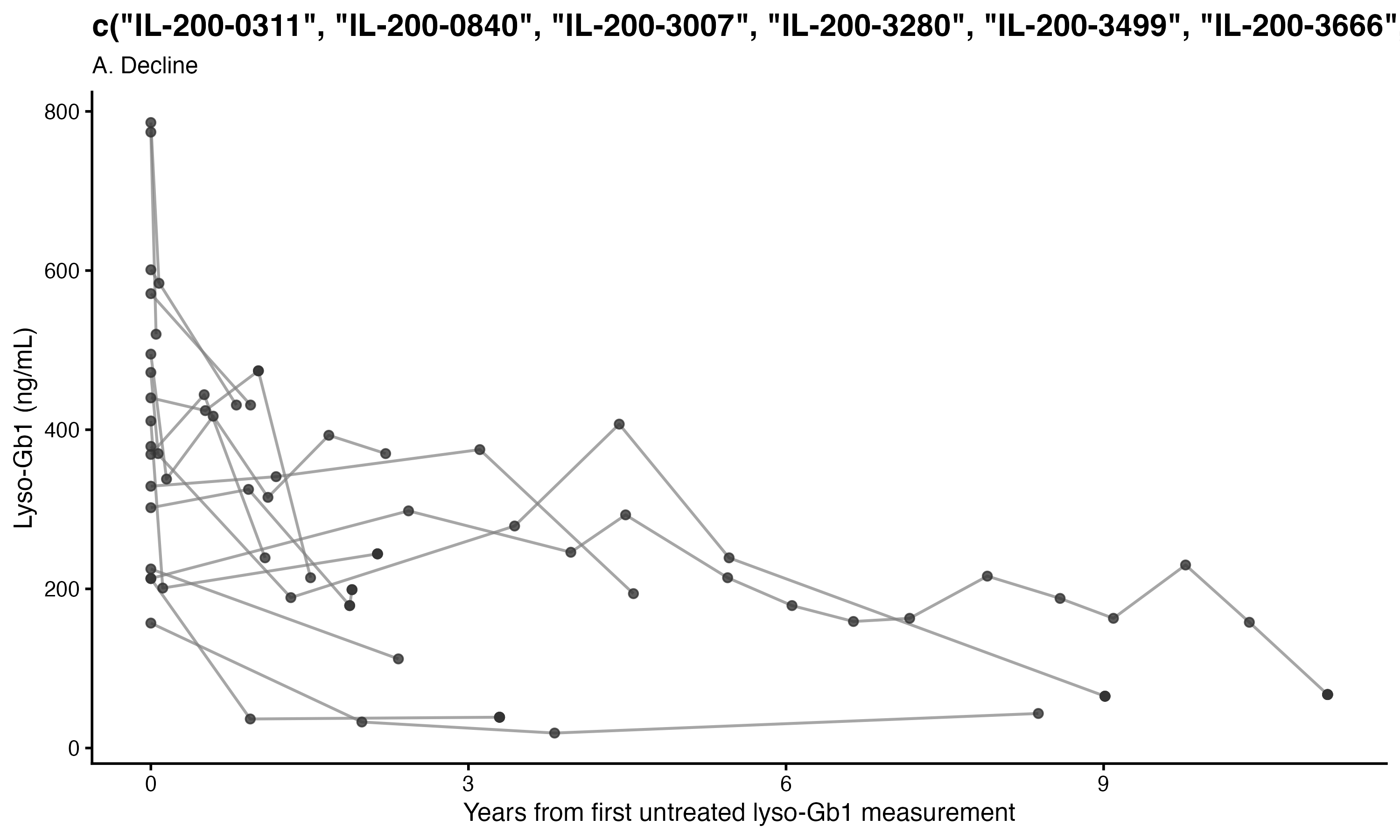

Supplement: Supplementary file 1 [file ijms-27-03726-s001.zip › Figure S1A.png]

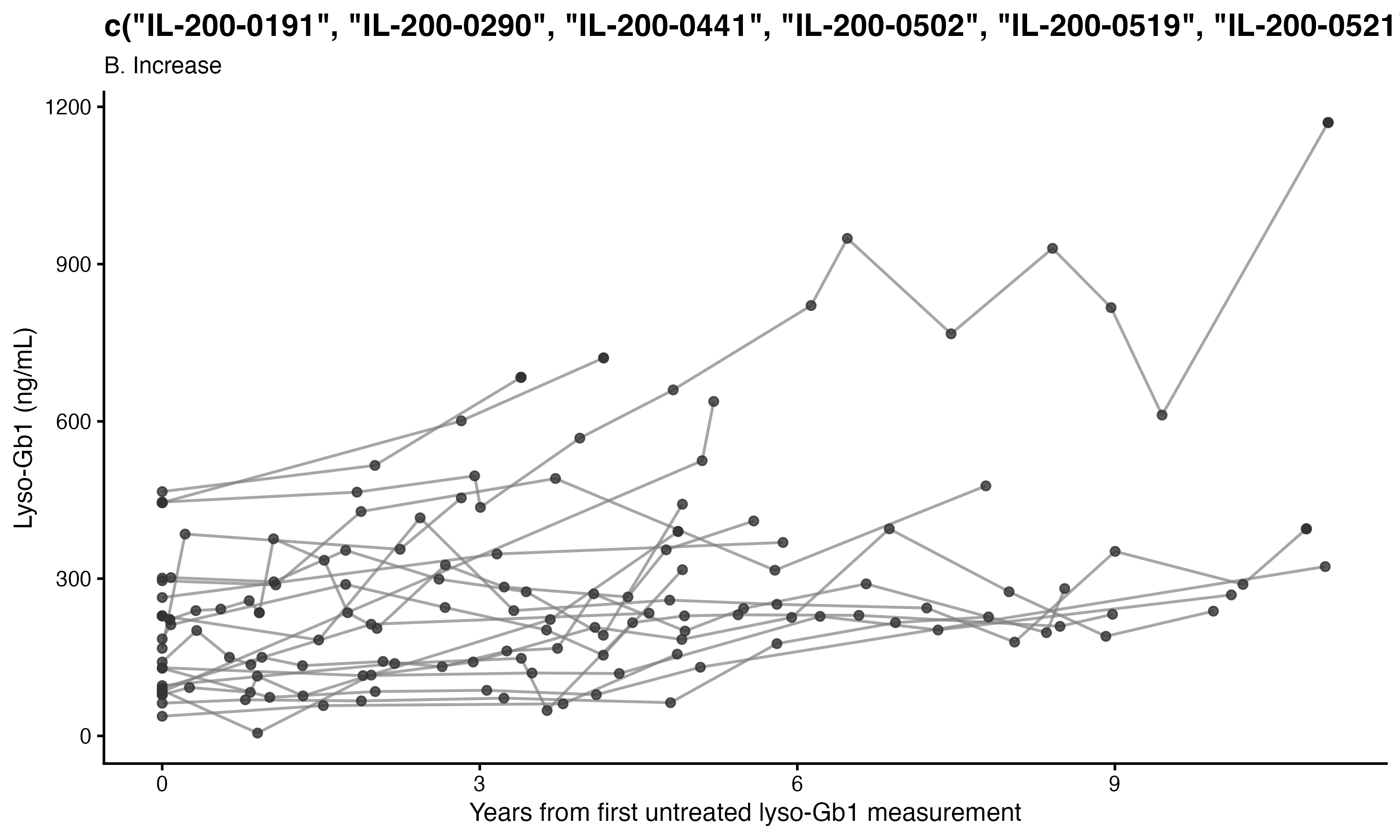

Supplement: Supplementary file 1 [file ijms-27-03726-s001.zip › Figure S1B.png]

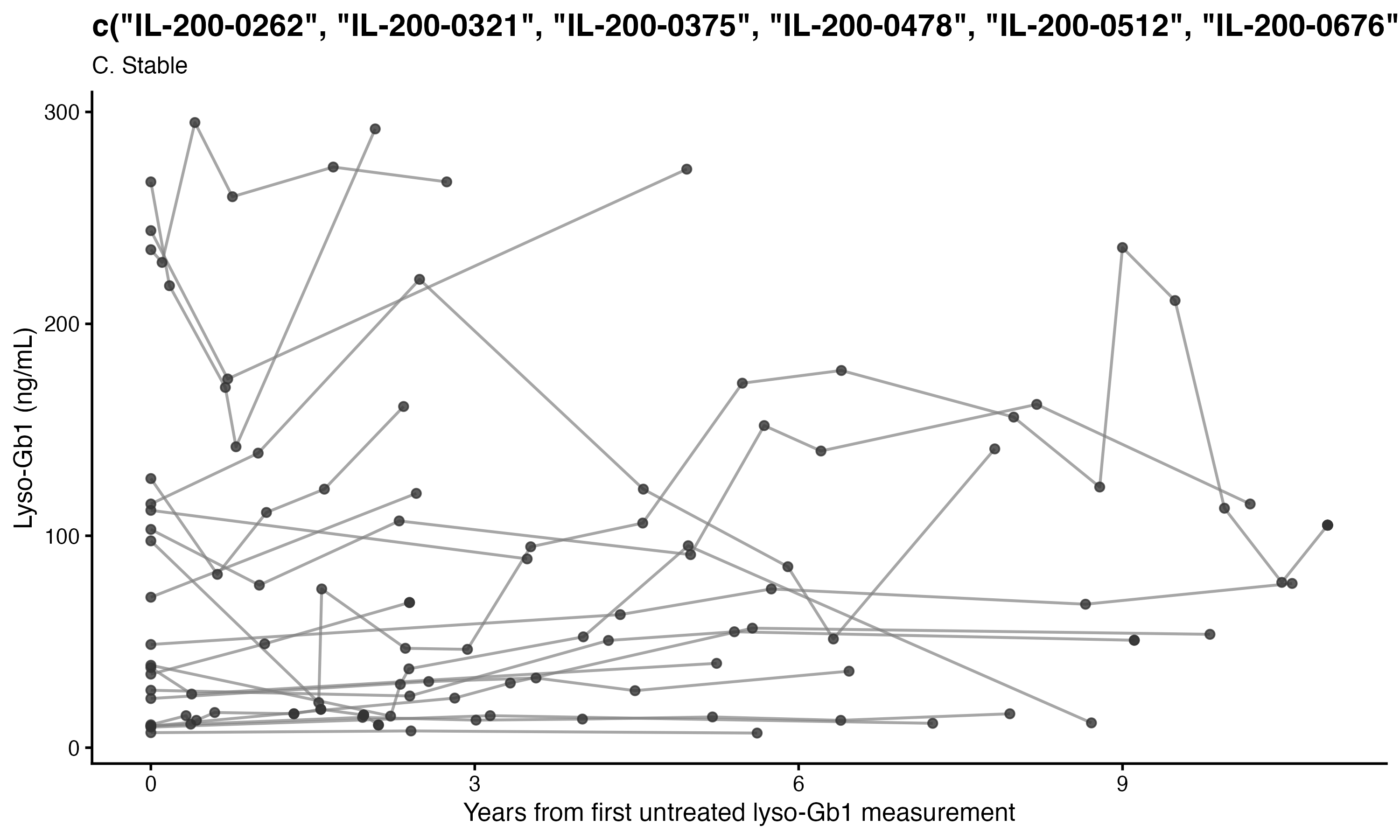

Supplement: Supplementary file 1 [file ijms-27-03726-s001.zip › Figure S1C.png]

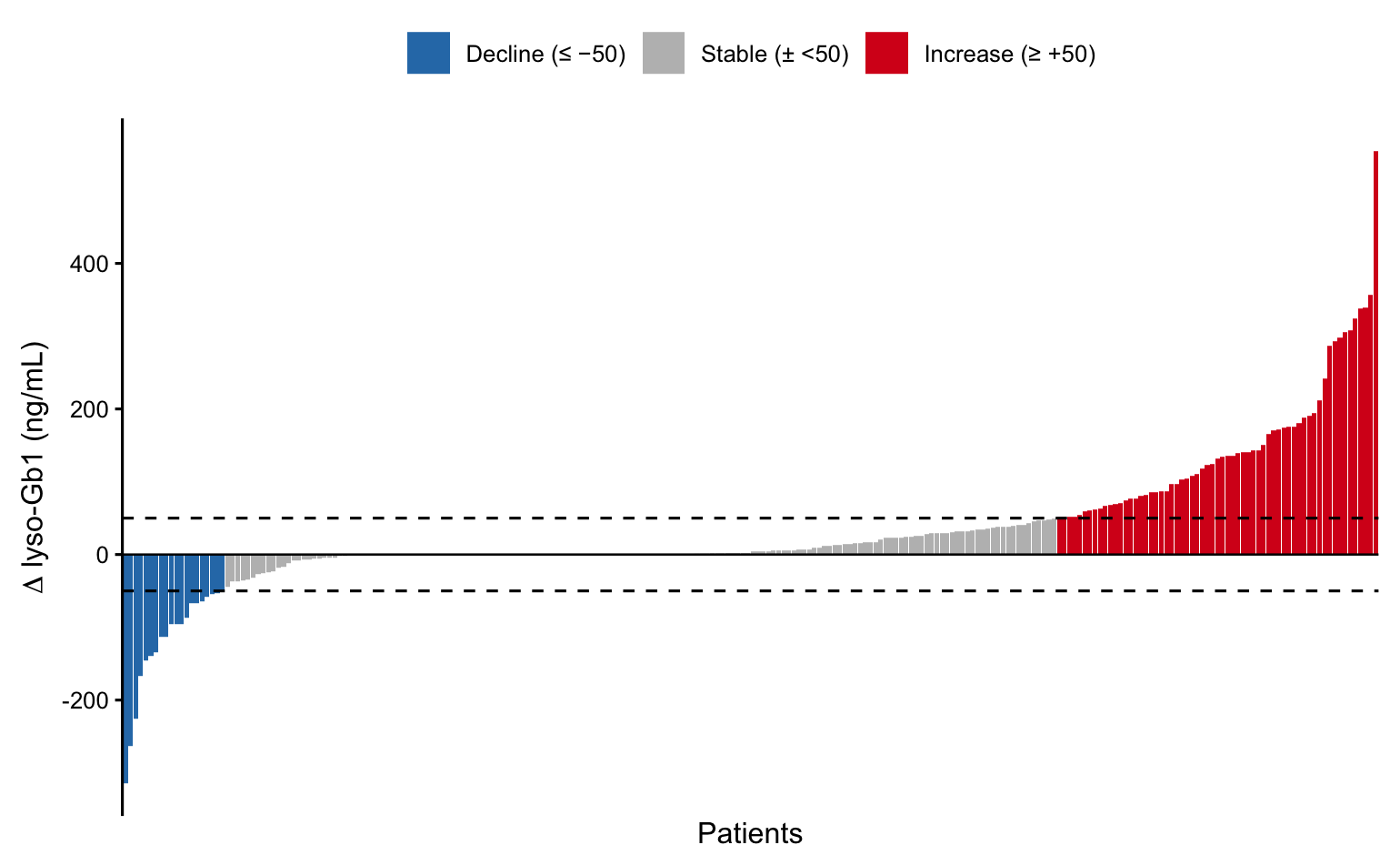

Supplement: Supplementary file 1 [file ijms-27-03726-s001.zip › Figure S2.png]
